# Causal contributions of human frontal eye fields to distinct aspects of decision formation

**DOI:** 10.1038/s41598-020-64064-7

**Published:** 2020-04-30

**Authors:** Carolina Murd, Marius Moisa, Marcus Grueschow, Rafael Polania, Christian C. Ruff

**Affiliations:** 1 Zurich Center for Neuroeconomics, Department of Economics, University of Zurich, Rämistrasse 71, Zurich, 8006 Switzerland; 2 Decision Neuroscience Lab, Department of Health Sciences and Technology, ETH Zurich, Rämistrasse 101, Zurich, 8092 Switzerland; 3 Present Address: Department of Penal Law, School of Law, University of Tartu, Teatri väljak 3, Tallinn, 10143 Estonia

**Keywords:** Decision, Perception

## Abstract

Several theories propose that perceptual decision making depends on the gradual accumulation of information that provides evidence in favour of one of the choice-options. The outcome of this temporally extended integration process is thought to be categorized into the ‘winning’ and ‘losing’ choice-options for action. Neural correlates of corresponding decision formation processes have been observed in various frontal and parietal brain areas, among them the frontal eye-fields (FEF). However, the specific functional role of the FEFs is debated. Recent studies in humans and rodents provide conflicting accounts, proposing that the FEF either accumulate the choice-relevant information or categorize the outcome of such evidence integration into discrete actions. Here, we used transcranial magnetic stimulation (TMS) on humans to interfere with either left or right FEF activity during different timepoints of perceptual decision-formation. Stimulation of either FEF affected performance only when delivered during information integration but not during subsequent categorical choice. However, the patterns of behavioural changes suggest that the left-FEF contributes to general evidence integration, whereas right-FEF may direct spatial attention to the contralateral hemifield. Taken together, our results indicate an FEF involvement in evidence accumulation but not categorization, and suggest hemispheric lateralization for this function in the human brain.

## Introduction

Numerous studies in humans and animals report that during perceptual decisions, sensory information presented over time can correlate linearly with increasing neural activity in frontal and parietal brain areas. While this has most often been investigated in the visual domain, similar results have also been found for other sensory modalities (e.g. auditory and tactile^1–4^, for review^5^). Neuronal activity in these frontal and parietal areas has been shown to gradually increase during stimulus presentation, as a function of the incoming evidence for one of the choice options^3,4,6,7^. Among these regions most consistently implicated in this situation are the primate FEFs^3,8–11^. While the FEFs are part of the oculomotor system, they have also been shown to contain neurons correlating with tactile decisions (e.g^12^.). These findings have led to proposals that the FEF – in addition to their putative role in generic attentional^11^ and motor-related processes^13,14^ – are directly involved in integrating sensory evidence over time to inform perceptual decisions^3,15,16^.

In apparent contrast to these proposals, optogenetic inactivation of the FOF (rodent homolog of human FEF^17^) during perceptual choices^4^ has been found to impair decision performance only when the inactivation is induced just some milliseconds prior to choice, while the integrated information is categorized into discrete choice-options (e.g. “go left” vs “go right”). Inactivation of the FOF during the preceding stimulus presentation and evidence integration had no effect on the animal’s choice behaviour. This was interpreted as evidence that the rodent FOF only support conversion of the outcome of the integration process into the categorical choice, rather than contributing to the actual information integration process (that may occur somewhere else in the brain). However, little is known about the specific functional contributions of the *human FEF* to these perceptual decision processes. This is because, for the human brain, most of the evidence for a FEF involvement in decision-making comes from brain imaging studies that cannot directly demonstrate whether FEF activity is indeed causally relevant for choice behaviour.

One way to determine the specific causal role of human FEF activity for perceptual decision formation is to employ non-invasive perturbation methods that enable temporary disruption of neural activity. One such method is TMS, which only has modest effects when compared to optogenetic inactivation used in animals, since the stimulated area is disrupted mildly rather than silenced (for review^18,19^). Nevertheless, TMS still enables inference on the causal relevance of the area for certain cognitive functions. For instance, several neurostimulation studies have demonstrated that the FEFs are generally involved in decisions about spatial stimulus features^20–22^ and target discrimination during visual search^23–26^. However, in most of these studies the focus was not on specific sub-processes of decision formation; the stimulation period often started with the onset of the stimulus and exceeded it in duration^21,23,25^, leaving it unclear whether the FEF contributions relate to temporally isolated sub-processes. Only a few studies have investigated the functionality of the FEF with more temporally restricted double-pulse TMS administered at different timepoints during stimulus presentation^22,24^. For example, in Bardi *et al*.^22^ the FEF was stimulated with double-pulse TMS at one of four possible timepoints during presentation of a classical Simon-effect stimulus. TMS over FEF reduced the Simon effect, but only when the stimulation was applied at certain timepoints. However, the stimulus in this study was presented unchanged for a very brief timeperiod (200 milliseconds); it is therefore unclear how different stimulation time-windows may relate to evidence integration and categorization sub-processes of decision formation. Finally, some of the cited TMS studies have demonstrated that behavioural effects of FEF-TMS on visual decisions are more pronounced for stimulation of one of the two hemispheres^20,22,25,27^. This was also found in Bardi *et al*.^22^, who showed that left FEF TMS reduced the Simon effect when applied at earlier timepoints, but that right FEF TMS reduced the Simon effect when applied at a later timepoint during stimulus presentation. No such lateralization was observed in rodent studies^4,7,28^, which already might suggest differences in the roles of human FEF and rodent FOF.

In the current study, we set out to determine whether the role of the human left and/or right FEFs in decision formation is to support evidence integration or categorization. For this purpose, we employed online double-pulse TMS (dTMS) to temporarily disrupt functionally relevant FEF activity at two different timepoints (early and late) during stimulus presentation, in a two-alternative forced choice (2AFC) perceptual decision-making task optimized for studying evidence integration over longer timescales. We used tactile stimuli instead of auditory stimulation to avoid interference of TMS-related click sounds with the task, as well as to circumvent  possible influences of TMS-related saccades and blinks during stimulus presentation. As TMS can have task- and timing-dependent non-neural side effects due to the tactile sensation and the sound of the TMS pulse^29^, it is necessary to have a control for these effects. Therefore, to reliably control for the non-neural side effects of TMS, we stimulated vertex as a control site using the same TMS-protocol and stimulation timepoints (early T1 and late T2) as in the active FEF-dTMS condition.

We hypothesized that if the FEF supports the integration of decision evidence, then early disruption of the FEF (during evidence presentation; TMS timing T1) should impair task performance, but late disruption (after most of the evidence has been presented and choices need to be categorized; TMS timing T2) should have no effect. However, if the FEF only contributes to the categorization process (as suggested in rodents^4,28^), then only late but not early TMS disruption should lead to impaired performance.

## Materials and Methods

### Participants

Forty-three volunteers participated in two experimental sessions: the fMRI localizer and TMS session (left FEF group: 11 females, 12 males; M_age_ = 24.8, SD_age_ = 3.7; right FEF group: 11 females, 9 males; M_age_ = 20.9, SD_age_ = 1.9). All participants gave written informed consent and all procedures were approved by the Research Ethics Committee of the Canton of Zurich and all methods were performed in accordance with the internationally accepted guidelines^30,31^. All participants were healthy, well-rested, right-handed, had normal vision and no contraindication to fMRI or TMS. For their participation, they received a participation fee of CHF 44/hour.

### Participant exclusion

Participants were excluded in two stages of the study. We initially scanned thirty subjects per stimulation group, but five subjects per group (10 in total) had to be excluded after the fMRI localizer session due to either artefacts in the imaging data or inability to perform the task to a satisfactory level (at least 65% accuracy for the smallest difference in the number of pulses level, i.e. −2/+2). At the beginning of the TMS session, two more participants from the first group (left FEF group) and five from the second group (right FEF group) had to be excluded due to TMS-related issues – in these participants, the relatively high stimulation intensity used (110% of their individual resting motor threshold - rMT) caused face or scalp muscle contractions or eyelid-twitches that were disturbing and prevented participants from focusing on the tactile task. Thus, these side effects of the stimulation must be treated with care particularly when TMS is applied online with the task. This resulted in the final sample size of 43 subjects.

### Experimental design and statistical analyses

All subjects performed the same behavioural task twice, first during the fMRI session and then during the TMS session.

### Behavioural task

**Figure 1:**
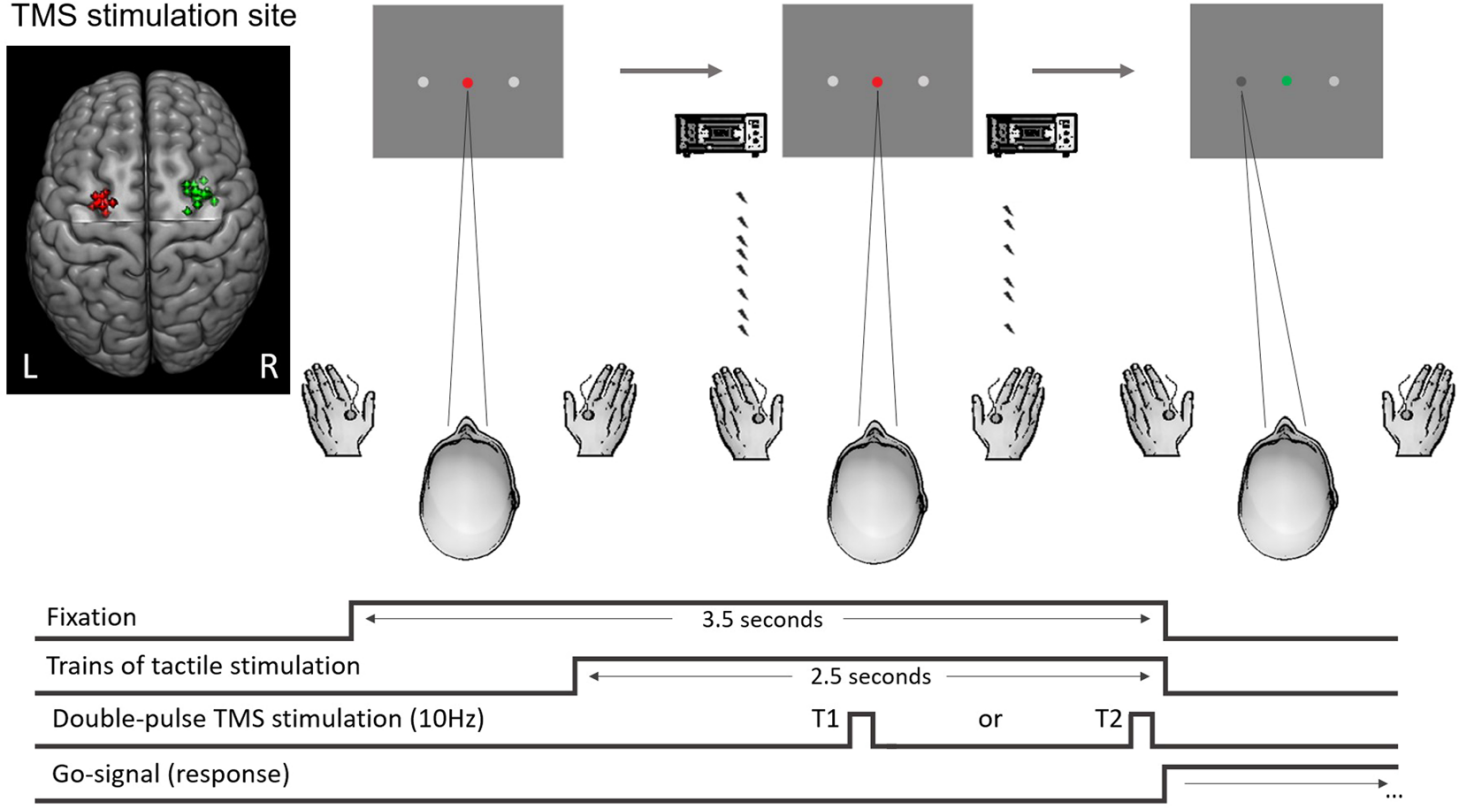
Behavioural task. Each trial started with the appearance of a red fixation point and gray response dots on either side of the fixation. One second after the onset of the trial, the simultaneous trains of randomly-timed tactile pulses were presented to both hands for 2.5 seconds. Once the trains stopped, the fixation point turned green to signal that participants should give their response by moving their gaze to the respective response dot (either left or right) displayed on the screen. The chosen response dot darkened when the response was registered. In the main experiment, the first pulse of the dTMS (10 Hz, 110% of rMT) was delivered either 1.25 seconds (T1) or 2.25 seconds (T2) into the tactile stimulation. The mean MNI coordinates across participants for the left FEF stimulation group were (−27; −4; 56), and for the right FEF stimulation group were (30; −1; 56). Individual stimulation sites are presented in the inset (red dots for the left and green dots for the right FEF stimulation group). MRIcroGL software was used (http://www.mccauslandcenter.sc.edu/mricrogl/) for visualization of the stimulation sites.

We used a tactile adaptation of a ‘Poisson-clicks’ accumulation task^4,28,32^ requiring participants to judge which of two simultaneous trains of randomly-timed tactile pulses (each train applied on either hand) contained more pulses. In this task, the perceived information (i.e., evidence) supporting either of the possible choice-options must be integrated over time to derive the final decision; which train contained a higher number of tactile stimuli (see Fig. 1). The same behavioural task was first performed inside an fMRI scanner so that we could identify the precise coordinates of the FEFs for each participant. These individual FEF coordinates were used in the main experiment as the target site of TMS-stimulation (see Fig. 1, left side inset).

### Visual display

In the TMS experiment, the subjects were presented with a three-dot visual display (1280 ×1024 pixels) containing a central fixation dot (size 0.2° visual angle) and two response dots (size 0.3°), one on either side of the fixation (Fig. 1). The distance between the fixation dot and each response dot was 5.6° and the viewing distance was 85 cm. The respective parameters during the fMRI localizer session were comparable (central fixation dot size 0.16°, response dot size 0.25°, response dot distance from central fixation 4.7°, viewing distance 126.5 cm). Each trial started with the fixation dot turning red so that subjects could fix their gaze on the central fixation dot without blinking and could pay attention to the upcoming tactile stimuli (see below). The end of the tactile stimulation was indicated by the central fixation dot turning green, which informed subjects that they should give their response by moving their gaze to the response dot on the side corresponding to the hand that was stimulated more often during this train (left or right). As the response was recorded, the selected response dot darkened for 300 msec before the central fixation dot turned white to signal the inter-trial-rest, during which subjects could blink or briefly rest their eyes. Participants’ responses were recorded with an eye-tracking camera with 500 Hz sampling rate (EyeLink 1000, SR Research Ltd., Canada).

### Tactile stimulation

In each trial, subjects were presented with two simultaneous trains of randomly timed tactile stimuli (pulses) – one train on their left and the other train on their right hand. The tactile stimulation lasted for 2.5 sec. Subjects were instructed not to count each pulse numerically, but to assess on which hand they had felt more pulses. The tactile stimulation was presented via two current stimulators (DS5 Isolated Bipolar Current Stimulator, Digitimer Ltd., UK), and the pulses were adjusted so that the intensity on the left and right hand felt subjectively equal (to avoid subjects comparing the perceived strength instead of the number of stimuli received). The duration of one pulse was 2 msec and the total number of the tactile stimuli presented on the left and right hand was set at constant: #Right + #Left = 14 pulses in 2.5 sec. The timing of the pulses on each hand was generated as a poisson-train via the Matlab function poissrnd with lambda set to 5.6 pulses per second. To keep the total number of tactile pulses constant, for each trial the function was repeated until the number of resulting random timings matched the number of #Right and #Left pulses in the respective trial. The difference in number of pulses (#Right − #Left) had seven possible levels (−6; −4; −2; 0; +2; +4; +6 pulses) during the fMRI session. However, as there were no performance differences in terms of accuracy or the response times for the two highest levels of the difference in number of pulses (#Right - #Left values −6 vs −4, and values +4 and +6), the TMS sessions only presented five levels of pulse differences (−4; −2; 0; +2; +4 pulses). Our subjects were not informed about the total number of pulses nor of the levels of pulse differences.

As it was important for our task that all stimuli presented in trains would be perceived and discriminated, the inter-pulse-interval (IPI) was set so that the minimum IPI for consecutive pulses on the same location (same hand) was 130 msec. This minimum IPI exceeded the somatosensory temporal discrimination threshold (STDT) of 15–80 msec found in other studies^33–37^. The minimum IPI between consecutive pulses between sides (left hand and right hand) was 65 msec, which exceeds the just noticeable difference (JND) and simultaneity judgment (SJ) threshold levels ranging from 12.5 and 49 msec in different studies^34,38–41^. This prevented subjects from pairing the pulses based on subjectively perceived simultaneity. Only the two first pulses of both trains were presented with an IPI of 0 msec so that they were perceived to be simultaneous. This ensured that responses were not biased by the earlier onset of one of the two trains, as previously shown in a similar experimental setup with auditory stimuli^32^. To confirm that our participants integrated evidence until the end of the tactile trains and did not make their choice at an earlier timepoint (e.g., based on the evidence difference during the first half of the trains), we ran a logistic regression model in which we regressed choices on two predictors: the evidence available in the first half of the trains and the final evidence at the end of the stimulus train. Both predictor variables were obviously correlated (Pearson r = 0.67, p < 0.001) but nevertheless had independent influences on choice, as reflected by statistically significant beta weights. The weight for the final evidence at the end of the stimulus train (*β* = 0.486, p < 0.001) was higher than the weight for the evidence in the first half of the stimulus trains (*β* = 0.065, p < 0.001) (t(42) = 11.173, p < 0.001, post-hoc paired t-test on betas). This confirms that participants accumulated evidence until the end of the tactile trains and based their choices on the final difference between both stimulus trains.

### Stimulation electrodes placement

The stimulation electrodes were placed on the first dorsal interosseous muscles (FDI) of both hands. This location was chosen for two main reasons: a) sensitivity of this region (i.e., stimulation intensity was similar for both hands) varied less between hands and between subjects than for example the sensitivity of the tip of the fingers, b) larger skin surface enabled more stable placement of stimulation electrodes (compared to e.g. the area of the tip of the finger).

### Intensity of tactile stimuli

Optimization of the intensity of tactile stimuli started with an initial intensity of 2 mA. This intensity was then gradually increased or decreased until the subject judged it to be non-painful but discriminable. The intensities were then slightly adjusted to ensure that subjects perceived the intensities to be equal between hands. Across all subjects, the mean intensity on the left hand was 2.1 mA (SD 1.3 mA) and on the right hand 2 mA (SD 1.6 mA).

### fMRI session

#### Procedure

To localize the individual coordinates of the stimulated FEF, in a first experimental session the participants performed the task inside the MR scanner. Each participant first practiced for 20 trials and then performed 264 trials that were divided between six runs. The order of trials with different evidence levels (difference in number of pulses) was randomized within runs. The inter-trial interval (ITI) was varied between 7 and 11 seconds (with mean ITI of 8.5 seconds). Eight null-event trials (without tactile events) were presented during each run.

#### fMRI acquisition

Functional imaging was performed on a Philips Achieva 3 T whole-body MR-scanner equipped with an eight-channel MR head coil. In total, we conducted 6 experimental runs, each of which contained 206 volumes (voxel size = 3 × 3 × 3 mm^3^, 0.5 mm gap, matrix size = 80 × 80, TR/TE = 2200/30 msec, flip angle = 90, parallel imaging factor =1.5, 36 slices acquired in ascending order for full coverage of the brain). We also acquired T1-weighted multi-slice fast-field echo B0 scans that were used for correction of possible geometric distortions due to magnetic field inhomogeneities (voxel size = 3 × 3 × 3 mm^3^, 0.5 mm gap, matrix size = 80 × 80, TR/TE1/TE2 = 452/4.3/7.4 msec, flip angle = 44, no parallel imaging, 40 slices). Additionally, we acquired a high-resolution T1-weighted 3D fast-field echo structural scan used for image registration during post-processing (181 sagittal slices, matrix size = 256 × 256, voxel size = 1 mm^3^, TR/TE/TI = 8.3/3.9/181 msec).

#### fMRI analysis

The fMRI data were analysed with Statistical Parametric Mapping (SPM8, http://www.fil.ion.ucl.ac.uk/spm) implemented in Matlab (MathWorks, Natick, Massachusetts, U.S.A). Pre-processing of the functional time series included motion correction, slice time correction, normalization to Montreal Neurological Institute (MNI) space, spatial resampling to 3 mm isotropic voxels, temporal high-pass filtering and spatial smoothing (Gaussian with 8 mm full-width at half-maximum).

Statistical analysis followed a two-stage procedure. First, we computed a single-subject fixed-effects model for each participant by multiple regression of the voxel-wise time series onto a composite model containing the covariates of interest. To identify the precise location of the FEF site involved in the decision, the GLM design matrix included our main regressor of interest which modelled the response of the participants as epochs of duration corresponding to the response time. The model also contained an indicator of the task difficulty (parametric modulator with 4 difficulty levels: 6, 4, 2 and 0) as well as the subjective response side (parametric modulator with 2 levels, 1 for response to the right and −1 for responses to the left; see Behavioural task).

In addition, we also modelled the following regressors of no interest: an indicator of the missed responses, 6 motion parameters (to account for BOLD signal changes that correlated with head movements), and indicator functions for blinks, saccades, and pupil activity. The last two regressors additionally contained a parametric modulation with saccade size and pupil size, respectively. We removed possible geometric distortions using the “unwarp” toolbox implemented in SPM8, by means of subject-specific field-maps. To allow for population inference, we fed the individual contrast images into second-level random-effects analyses. The group activation map corresponding to the task-related decision process (response) was thresholded at p < 0.05 FWE corrected for multiple comparisons at the cluster level (with cluster-forming threshold p < 0.001). This map was masked by standard FEF ROIs generated by a meta-analysis (term ‘frontal eye’, Neurosynth database dated 30th of January 2016, http://neurosynth.org/). Within this general FEF mask, we determined the individual activation peak as the stimulation site.

#### fMRI session behavioural analysis

The proportion of rightward choices revealed a main effect of evidence (F (6,252) = 801.01, p < 0.001, ${\eta }_{p}^{2}$ = 0.95, repeated measures ANOVA), indicating that participants were sensitive to task difficulty and made increasingly more rightward responses when more pulses were presented on the right hand.

### TMS session

In the beginning of the TMS session, we first determined the resting motor threshold (rMT) in each participant by stimulating M1 in the left hemisphere. We defined the rMT as the percent of maximum stimulator output (mean intensity was 51% (SD 8%)) required to elicit motor evoked potentials with an amplitude greater than 200 μV in five times out of ten pulses. TMS-system Magstim Rapid2 Plus (Magstim, UK) and TMS Neuronavigation system (BrainSight 2, Rogue Research Inc., Canada) were used for stimulation. The intensity of the double-pulse TMS (dTMS) was set at 110% of rMT, with the frequency of 10 Hz (i.e. the second pulse was presented 100 msec after the first pulse).

#### Procedure

During the TMS session, each participant first practiced for 20 trials and then performed 480 trials that were divided between eight runs (four control runs with vertex-dTMS and four runs with FEF-dTMS, the order of the runs was counterbalanced between subjects). In each run, during half of the trials the TMS was presented in the middle of the tactile stimulation trains (early stimulation time T1, during the evidence integration period of decision formation) and in half of the trials during the last 250 msec of the tactile stimulation trains (late stimulation time T2, the categorization stimulation). The order of trials with different TMS timing and the level of difference in number of pulses was randomized within runs. Inter-trial-interval was 7.5 sec.

#### Site localization

The individually-determined FEF sites (see fMRI analysis subsection and Fig. 1) were marked on the individual structural MRI scans, and the neuro-navigation system was used to co-register each participant with their structural MRI.

#### Statistical analysis

Statistica 13.0 (Dell Software) was used for all analyses. We conducted analysis of variance (ANOVA) and post-hoc paired t-tests (detailed analysis under ‘Results’ section). We ran an overall four-way repeated measures ANOVA on the proportion of rightward responses (group × TMS × TMS timing × evidence) (see Table 1). As a follow-up analysis, we ran separate ANOVAs per stimulation groups (three-way repeated measures ANOVAs) (see Tables 2 and 3) and, to specify the nature of TMS effect on the task performance in both stimulation groups, two-way ANOVAs (TMS × evidence) with logistic link function separately for the timepoints (T1 and T2) (see Tables 4 and 5). For the two-way ANOVAs we also conducted post-hoc paired two-tailed t-test on the slopes and biases of the sigmoid curves for active FEF-dTMS and control vertex-dTMS conditions. The p-values were corrected for multiple comparisons.

**Table 1:** Four-way repeated measures ANOVA on the proportion of rightward responses across stimulation groups (left FEF and right FEF stimulation).

Effect	SS	df	MS	F	p	${{\boldsymbol{\eta }}}_{{\boldsymbol{p}}}^{{\boldsymbol{2}}}$
Group	0.217	1	0.217	2.035	0.161	0.047
Error	4.363	41	0.106			
TMS	0.001	1	0.001	0.091	0.765	0.002
TMS × group	0.014	1	0.014	1.254	0.269	0.030
Error	0.459	41	0.011			
TMS timing	0.00	1	0.001	0.025	0.876	0.001
TMS timing × group	0.006	1	0.006	1.070	0.307	0.025
Error	0.228	41	0.006			
evidence	68.496	4	17.124	600.236	0.001**	0.936
evidence × group	0.154	4	0.038	1.346	0.255	0.032
Error	4.679	164	0.028			
TMS × TMS timing	0.022	1	0.021	2.955	0.093	0.067
TMS × TMS timing × group	0.020	1	0.020	2.739	0.105	0.063
Error	0.298	41	0.007			
TMS × evidence	0.055	4	0.014	1.768	0.138	0.041
TMS × evidence × group	0.042	4	0.011	1.361	0.250	0.032
Error	1.275	164	0.008			
TMS timing × evidence	0.047	4	0.012	1.215	0.306	0.029
TMS timing × evidence × group	0.034	4	0.009	0.881	0.477	0.021
Error	1.575	164	0.010			
TMS × TMS timing × evidence	0.027	4	0.007	0.874	0.481	0.021
TMS × TMS timing × evidence × group	0.077	4	0.019	2.460	0.047*	0.057
Error	1.284	164	0.008			

^*^p < 0.05; **p < 0.001; ***p < 0.0001.

**Table 2:** Three-way repeated measures ANOVA on the proportion of rightward responses across stimulation timings, the left FEF stimulation group.

Effect	SS	df	MS	F	p	${{\boldsymbol{\eta }}}_{{\boldsymbol{p}}}^{{\bf{2}}}$
TMS	0.004	1	0.004	0.296	0.592	0.013
Error	0.299	22	0.014			
TMS timing	0.002	1	0.002	0.347	0.562	0.016
Error	0.146	22	0.007			
evidence	38.808	4	9.702	343.247	0.001**	0.940
Error	2.487	88	0.028			
TMS × TMS timing	0.000	1	0.000	0.002	0.964	0.000
Error	0.169	22	0.008			
TMS × evidence	0.054	4	0.014	1.717	0.153	0.072
Error	0.692	88	0.008			
TMS timing × evidence	0.076	4	0.019	1.752	0.146	0.074
Error	0.953	88	0.011			
TMS × TMS timing × evidence	0.082	4	0.021	2.475	0.049*	0.101
Error	0.730	88	0.008			

^*^p < 0.05; **p < 0.001; ***p < 0.0001.

**Table 3:** Three-way repeated measures ANOVA on the proportion of rightward responses across stimulation timings, the right FEF stimulation group.

Effect	SS	df	MS	F	p	${\eta }_{p}^{2}$
TMS	0.011	1	0.011	1.260	0.276	0.062
Error	0.159	19	0.008			
TMS timing	0.004	1	0.004	0.853	0.367	0.043
Error	0.082	19	0.004			
evidence	30.426	4	7.607	263.809	0.001**	0.933
Error	2.191	76	0.029			
TMS × TMS timing	0.039	1	0.039	5.686	0.028*	0.230
Error	0.129	19	0.007			
TMS × evidence	0.044	4	0.011	1.434	0.231	0.070
Error	0.583	76	0.008			
TMS timing × evidence	0.010	4	0.002	0.283	0.888	0.015
Error	0.621	76	0.008			
TMS × TMS timing × evidence	0.026	4	0.007	0.897	0.470	0.045
Error	0.554	76	0.007			

^*^p < 0.05; **p < 0.001; ***p < 0.0001.

**Table 4:** Two-way ANOVA (with logistic link function) on the proportion of rightward responses, left FEF stimulation group.

Effect	df	Wald χ^2^	p
Evidence integration period (T1)			
evidence	4	1301.79	0.001**
TMS	1	0.082	0.774
TMS × evidence	4	13.09	0.012*
**Categorization period (T2)**			
evidence	4	1209.87	0.001**
TMS	1	0.355	0.551
TMS × evidence	4	2.39	0.664

^*^p < 0.05; **p < 0.001; ***p < 0.0001.

**Table 5:** Two-way ANOVA (with logistic link function) on the proportion of rightward responses, right FEF stimulation group.

Effect	df	Wald χ^2^	p
Evidence integration period (T1)			
evidence	4	1082.67	0.001**
TMS	1	4.612	0.032*
TMS × evidence	4	3.613	0.461
**Categorization period (T2)**			
evidence	4	1057.29	0.001**
TMS	1	0.836	0.360
TMS × evidence	4	3.12	0.538

^*^p < 0.05; **p < 0.001; ***p < 0.0001.

#### Comparison of fMRI session and TMS session control condition

**Figure 2:**
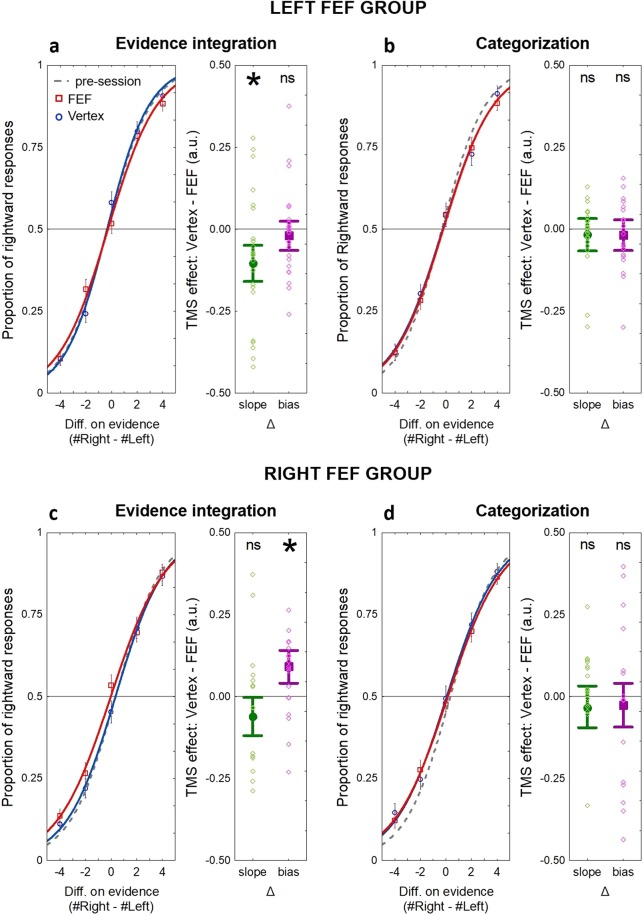
The proportion of rightward choices as a function of the signed difference in evidence (tactile pulses) across the stimulation group. (**a**) TMS effect on choices during evidence integration period (T1) in the left FEF group. Left FEF stimulation resulted in decreased sensitivity to evidence, reflected by the significant difference between active (FEF-stimulation) and control (vertex-stimulation) conditions *(∆ slope*). (**b**) TMS effect on choices during categorization period (T2) in left FEF group. There was no significant change in choice behaviour following FEF vs vertex stimulation during categorization period. (**c**) TMS effect on choices during evidence integration period (T1) in the right FEF group. Right FEF stimulation resulted in response bias towards rightward choices, reflected by a significant *∆ bias*. (**d**) TMS effect on choices during categorization period (T2) in right FEF group. There was no significant change in response behaviour following FEF vs vertex stimulation during categorization period. The dashed lines mark the performance in pre-session inside the MR scanner to illustrate the non-neural effects of TMS^29^ [see the Experimental design and statistical analyses section]. On the left-side figures (main plots), the dots present the data, while lines are logistic curves. The right-side figures represent the estimation of difference between the slope and bias of the two curves in the respective main plot (a, b, c or d), while dots represent individual data. Vertical bars on all plots denote +/− standard error of the mean.

To test the stability of the performance during different sessions, and control for possible non-neural side effects of TMS, we ran two-way ANOVAs with logistic link function on the performance in pre-session (fMRI) and the vertex-dTMS conditions of the TMS experiment. The performance in the vertex-dTMS condition and the performance in the pre-session were comparable when the stimulation took place earlier during the trial (T1) (left stimulation group: session (pre-session vs vertex-dTMS condition) × evidence interaction p < 0.61; right stimulation group: session (pre-session vs vertex-dTMS condition) × evidence interaction p < 0.16) (see Fig. 2a,c). As there was no significant effect, it seems that the subjects’ performance was stable over the session. However, the performance in vertex-dTMS condition during the later stimulation time point (T2) differed from the performance in the pre-session (see Fig. 2b,d) (left stimulation group: session (pre-session vs vertex-dTMS condition T2) × evidence interaction (p < 0.02); right stimulation group: session (pre-session vs vertex-dTMS condition T2) × evidence interaction p = 0.009). These results indicate a possible time-dependent non-neural side effect of TMS^29^, and further support the rationale of separate data analysis for the two stimulation timepoints (T1 and T2).

#### Data exclusion

Trials where blinks or eye movements broke fixation (deviation from central fixation dot over 2.4° either in horizontal and vertical direction) were excluded. In addition, runs during which participants reported the loss of sensitivity of the tactile stimuli on one hand were discarded (10 out of 344 runs across 43 participants). Trials with response times exceeding 2 standard deviations from the mean (within each participant) were categorized as outliers/missed trials and were excluded from the analysis. The response time-based exclusion was 6.25% of all trials (varying from 4.05% to 10.6% between participants). The overall trial exclusion was 12.8% of all trials.

## Results

For the main TMS experiment, we first assessed the general impact of the presented perceptual evidence and of TMS in a comprehensive, integrated analysis (repeated-measures ANOVA: group (left FEF vs right FEF) × TMS (active FEF vs control vertex) × TMS timing (T1 vs T2) × evidence (#Right pulses - #Left pulses; 5 levels)) (see Table 1). We found that participants were indeed sensitive to the signed difference in presented evidence, since they made increasingly more rightward choices the more tactile pulses were presented on the right versus left hand (main effect evidence: F (4, 164) = 600.24, p < 0.001, ${\eta }_{p}^{2}$ = 0.936, ANOVA) (see Table 1). However, this dependency of choice on the degree of evidence was differentially affected by the different types of TMS (four-way interaction stimulation group × TMS × TMS timing × evidence: F (4, 164) = 2.46, p = 0.047, ${\eta }_{p}^{2}\,$= 0.057, ANOVA) (see Table 1). To characterize the precise differences in effects for left- and right-FEF TMS administered at different time-points, we conducted three-way (see Tables 2 and 3) and two-way ANOVAs separately for the two stimulation groups (see Tables 4 and 5).

For both left- and right-FEF TMS, we found differences in stimulation effects between the different timepoints, which is in line with the assumption that left and right FEF might make different functional contributions during different stages of the decision process. For left-FEF stimulation, the TMS effects (difference between active and control TMS) slightly varied with both timing and evidence level (three-way interaction TMS × TMS timing × evidence (F (4, 88) = 2.48, p = 0.049, ${\eta }_{p}^{2}\,$= 0.101, ANOVA) (see Table 2), whereas for right-FEF TMS, these effects only varied with timing but were similar across evidence levels (two-way interaction TMS × TMS timing: F (1, 19) = 5.686, p = 0.028, ${\eta }_{p}^{2}\,$= 0.23; three-way interaction: F(4, 76) = 0.897, p = 0.47, ${\eta }_{p}^{2}$ = 0.045, ANOVA) (see Table 3).

We further specified the nature of these differences between effects for left- versus right-FEF TMS by means of two-way ANOVAs (TMS × evidence) with logistic link functions, conducted separately for the different timepoints (T1 and T2) (see Fig. 2). In both left and right FEF groups, stimulation only affected performance when FEF was disrupted during the evidence integration period (T1) but not during categorization (T2). However, in the *left FEF* stimulation group, this timepoint-specific effect reflected *decreased sensitivity to the presented evidence*, whereas in the *right FEF* group, it resulted in a *bias towards rightward choices*. The time-point-specific decreased sensitivity was demonstrated for the left FEF group by an interaction between TMS × evidence (Wald χ^2^ (4) = 13.09, p = 0.012, ANOVA) (Fig. 2a) and a shallower slope of the sigmoid curve (active FEF-dTMS vs control vertex-dTMS condition, t(22) = −2.368, p = 0.027, post-hoc paired t-test) (Fig. 2a) (Table 4). Although modest in size, these TMS effects were only expressed for left-FEF stimulation, as right-FEF stimulation did not lead to a general change in sensitivity to choice-relevant information (interaction TMS × evidence: Wald χ^2^ (4) = 3.613, p = 0.461, ANOVA; the slope of the sigmoid curve for active FEF-dTMS vs control vertex-dTMS condition, t(19) = −0.476, p = 0.64, post-hoc paired t-test) (Table 5). This pattern indicates that participants made more erroneous choices after left-FEF stimulation when perceptual evidence favoured one option over the other (irrespective of which side contained more stimuli).

TMS applied over the left FEF did not cause a bias towards right- or leftward responses, neither when this was tested across all trials (the bias of the curves in active FEF-dTMS vs control vertex-dTMS condition (t(22) = −0.691, p = 0.497, post-hoc paired t-test) nor when inspected only for trials without clear evidence in either direction (the proportion of rightward responses t(22) = 1.702, p = 0.103, post-hoc paired t-test). By contrast, in the right FEF stimulation group, TMS applied during evidence integration led to a bias towards rightward choices (main effect of TMS: Wald χ^2^ (1) = 4.612, p = 0.032, ANOVA; the bias of the sigmoid curves in active FEF-dTMS vs control vertex-dTMS condition (t(19) = 2.068, p = 0.05, post-hoc paired t-test) (Fig. 2c). This TMS-induced bias was again modest in size but evident only for right-FEF TMS (the bias in left FEF group vs right FEF group t(41) = −2.151, p = 0.037, post-hoc independent samples t-test), also when analysing only the trials without any clear evidence for either response option (the proportion of rightward responses in FEF-dTMS vs control vertex-dTMS condition t(19) = 2.671, p = 0.015, post-hoc paired t-test). No FEF-specific effects were found in neither stimulation group when the stimulation was applied during the categorization period (T2) (p > 0.36) (Fig. 2b,d), suggesting that - unlike in rodent studies – the human FEFs do not support choice categorization in the final stages of perceptual decision formation (Tables 4 and 5).

### Response times

We also measured response times to see whether the stimulation of the FEF affected the time needed to give responses. As in the task performance analysis described above, we performed overall four-way ANOVA (group × TMS × TMS timing × evidence) on mean response times (see Table 6). While participants gave faster responses when the difference between the tactile pulses presented on the hands was higher (main effect evidence: F (4, 164) = 11.94, p < 0.001, ${\eta }_{p}^{2}\,$= 0.225, ANOVA) and when dTMS was applied later during the stimulus presentation (main effect TMS timing: F (1, 41) = 52.21, p < 0.001, ${\eta }_{p}^{2}\,$= 0.56, ANOVA), there were no FEF-specific TMS effects on response times (see Table 6). Therefore, we did not conduct any further analysis on response times. As we did not have a free reaction time task, and since we emphasized choice accuracy over response speed, these results are not surprising and are fully in line with the lack of reaction time effects reported by comparable studies in rodents^4,7,28^.

**Table 6:** Four-way repeated measures ANOVA on response times across stimulation groups (left FEF and right FEF stimulation).

Effect	SS	DF	MS	F	P	${{\boldsymbol{\eta }}}_{{\boldsymbol{p}}}^{{\bf{2}}}$
group	0.007	1	0.007	0.067	0.797	0.002
Error	4.305	41	0.105			
TMS	0.000	1	0.000	0.000	0.991	0.000
TMS × group	0.005	1	0.005	1.009	0.321	0.024
Error	0.215	41	0.005			
TMS timing	0.116	1	0.116	52.209	0.001**	0.560
TMS timing × group	0.001	1	0.001	0.532	0.470	0.013
Error	0.091	41	0.002			
evidence	0.070	4	0.017	11.937	0.001**	0.225
evidence × group	0.010	4	0.003	1.699	0.153	0.040
Error	0.240	164	0.002			
TMS × TMS timing	0.002	1	0.002	3.298	0.077	0.074
TMS × TMS timing × group	0.001	1	0.001	0.496	0.485	0.012
Error	0.023	41	0.001			
TMS × evidence	0.005	4	0.001	1.327	0.262	0.031
TMS × evidence × group	0.005	4	0.001	1.299	0.272	0.031
Error	0.147	164	0.001			
TMS timing × evidence	0.001	4	0.001	0.297	0.880	0.007
TMS timing × evidence × group	0.002	4	0.001	0.578	0.679	0.014
Error	0.130	164	0.001			
TMS × TMS timing × evidence	0.001	4	0.001	0.127	0.972	0.003
TMS × TMS timing × evidence × group	0.006	4	0.002	1.953	0.104	0.045
Error	0.126	164	0.001			

^*^p < 0.05; **p < 0.001; ***p < 0.0001.

## Discussion

A recent paper^42^ has emphasized three criteria to determine whether a brain region is part of a causal neural circuit underlying evidence integration during decision formation: 1) inactivation of the region should affect task performance, 2) temporally precise interference of its activity during the integration step of decision formation should affect the performance, and 3) the graded value of the integrator or accumulator should be encoded in the neural activity of this region.

In the current study, we found corresponding stimulation-related effects on choice accuracy: disrupting the FEF during evidence integration, but not during the categorization period, affected task performance. These findings appear consistent with the criteria mentioned above, but they also indicate some interesting differences with recent rodent studies^4,28^. In these studies, inactivation of the FEF impaired task performance only when it was induced during the short categorization period, not when it was administered during evidence integration. Second, in our study (but not the rodent studies), the effects induced by FEF stimulation differed between the two hemispheres: Stimulation of the left FEF resulted in choice impairment that depended on the degree of choice evidence, suggesting that in humans, the left FEF might be causally involved in evidence integration process. In contrast, right FEF stimulation caused a slight shift in bias towards ipsilaterally presented stimuli. Such ipsilateral bias is consistent with commonly-reported shifts in spatial attention as a consequence of lesions of, or interference with, right-hemisphere parietal and frontal brain structures^11,25,27,43–47^.

The inconsistency between our results and the animal data – in terms of both causal involvement of the FEF in evidence integration vs categorization and hemispheric asymmetry in effects – may possibly reflect interspecies differences in human FEF versus rodent FOF. Despite parallel findings of graded activity increase in both these areas during perceptual choices^3,8,9,11,48^, the few studies directly comparing functional overlap across different species point to possible differences. For instance, comparative fMRI studies in humans and monkeys have shown weaker contralateral bias in fronto-parietal activity and stronger ipsilateral connections and larger hemispheric differences in connections in humans^49,50^. The latter set of findings appears consistent with the hemispheric differences in the effect of FEF stimulation observed here. Further support for lateralization of functions comes from human lesion studies. For example, hemi-spatial neglect (an inability to attend to the contralateral side) has been more frequently noted after right hemisphere lesions^51^. Similar attentional deficits have been found in several TMS studies^11,25,27,43–47^. In these studies, the effects on performance in attentional tasks following right hemisphere stimulation tend to be stronger and more extensive – often causing both ipsi- and contralateral effects –, while the effects of the left FEF stimulation seem to be more modest and bound to contralateral space^43–45^. Therefore, the right hemisphere is considered to be predominant in the top-down control of spatial attention.

Interestingly, lesion studies have also found that left-hemisphere (but not right-hemisphere) damage either decreases choice accuracy^52^ or increases response times^53^ in decision-making tasks. The predominant role of the left hemisphere in decisions is endorsed by imaging and TMS studies, with the former revealing that left-hemisphere frontal areas show stronger activity increases in response to changes in the amount of sensory evidence^8,54,55^. In congruence with these results, TMS studies have reported more erroneous choices or increase in response times for stimulation of left- compared to right-hemisphere frontal areas^56^. Moreover, TMS studies that have focused specifically on the functional role of the FEF have shown left-hemisphere dominance in spatial priming^21^, spatial conflict^22^ and conscious perception^57^. However, these previous studies, due to the implemented designs, could not reveal whether the observed behavioural effects reflect specific disruption of evidence integration processes or rather of other cognitive functions. Here, we demonstrated a temporally-specific involvement of the left FEF in the integration of incoming sensory evidence.

Clearly, our results cannot address whether the FEF in humans meets the third criterion described by Brody and Hanks^42^, i.e., whether this area carries out the integration process itself or influences the process remotely through its connections to other brain areas. To determine this, future studies should employ methods that allow temporally precise interference concurrently with recording of neural activity throughout the full brain network. Irrespective of these considerations, our findings indicate a causal link between activity in the left human FEF and a specific subprocess of human perceptual decision making. Our results may motivate further research into investigating how activity in this brain structure specifically interacts with other areas such as somatosensory cortices (SI/SII) and intraparietal sulcus (IPS) to jointly integrate choice evidence and transform it into actions.

## Data Availability

The datasets generated during and/or analysed during the current study are available from the corresponding author upon reasonable request.
